# Alterations of fecal microbiota and plasma metabolome in patients with Parkinson’s disease with rapid eye movement sleep disorder

**DOI:** 10.1128/msphere.00590-24

**Published:** 2025-05-09

**Authors:** Yangdanyu Li, Yuning Liu, Fujia Li, Xu Liu, Zixuan Zhang, Jinyu Li, Guiyun Cui, Chuanying Xu

**Affiliations:** 1 Department of Neurology, The Affiliated Hospital of Xuzhou Medical University, Xuzhou, China; University of Michigan-Ann Arbor, Ann Arbor, Michigan, USA

**Keywords:** Parkinson’s disease, rapid eye movement sleep behavior disorder, fecal microbiota, metabolome

## Abstract

Patients with Parkinson’s disease (PD) with probable rapid eye movement sleep behavior disorder (RBD) (PD-RBD), a specific subtype of PD, are characterized by the presence of more severe motor and non-motor symptoms. This study aimed to elucidate the characteristics and interactions of gut microbiota and plasma metabolic characteristics of PD-RBD, thus screening for the disease mechanisms. A total of 100 PD patients, 50 healthy controls (HCs) and 16 probable idiopathic RBD (iRBD) patients were collected. There were 33 PD-RBD and 67 patients without probable RBD (PD-nRBD) in PD patients. DNA extraction, PCR amplification, and high-throughput sequencing were used for intestinal microbiota analysis, and ultra-high liquid chromatography tandem mass spectrometry was used for metabolome analysis. Spearman analysis was applied to investigate the correlation of fecal microbiota and plasma metabolome. Our findings revealed *Lactobacillacea*e (*P* = 0.017), *Christensenellaceae* (*P* = 0.017), *Fusobacteriaceae* (*P* = 0.018), *Lactobacillus* (*P* = 0.035), *Christensenellaceae* R-7 group (*P* = 0.035), and *Fusobacterium* (*P* = 0.035) were significantly different in PD-RBD, PD-nRBD, and HC. Moreover, the differential metabolites identified in both PD-nRBD and PD-RBD were 3-hydroxy-2-methylpyridine-4,5-dicarboxylate (VIP = 5.802) and 3-methoxy-4-hydroxyphenylglycol sulfate (VIP = 5.732). Furthermore, our analysis revealed that 3-methoxy-4-hydroxyphenylglycol sulfate showed a positive correlation with *Lactobacillus* (*r* = 0.197, *P* = 0.049). Finally, functional analysis indicated that these distinctive microbiota and metabolites were primarily associated with phenylalanine metabolism and vitamin B6 metabolism. We managed to show that the differential microbiota, differential metabolites, and their interactions in PD-RBD compared to PD-nRBD and HC. This furthers our understanding of disease pathogenesis, and offers fresh perspectives on its detection and treatment.

**IMPORTANCE:**

There are currently fewer investigations on the intestinal microbiota and metabolites of probable rapid eye movement sleep behavior disorder (PD-RBD) and idiopathic RBD (iRBD). Our findings indicate that PD-RBD exhibits an increase in *Christensenellaceae* and 3-methoxy-4-hydroxyphenethyleneglycol sulfate, and that iRBD exhibits a similar trend. This suggests that the PD prodromal stage may have seen this alteration. Furthermore, functional analysis indicated that these distinctive microbiota and metabolites were primarily associated with phenylalanine metabolism and vitamin B6 metabolism. Basic experiments and multi-center, large-cohort clinical researches are worth conducting to confirm this, since they may offer insights for treating individuals with PD-RBD.

## INTRODUCTION

Parkinson’s disease (PD) is the second most prevalent neurodegenerative condition in the world. We discovered considerable variations in pathological alterations, clinical characteristics, disease progression, and treatment outcomes among distinct subtypes of patients with PD (1). And the uncertainty of treating each patient uniquely is increased by this heterogeneity. Recent studies have revealed that PD patients with probable rapid eye movement sleep behavior disorder (PD-RBD) have more severe mood changes, cognitive impairment, autonomic dysfunction, and more rapid motor progression (2, 3). Therefore, early identification and intervention are necessary for these patients.

However, the exact mechanism of the generation of PD-RBD is yet unknown, and it may be related to the brain-gut axis regulation (4), and neuroimmune metabolism (5). The “microbial-gut-brain axis” has been hypothesized by some researchers, and it aligns with the current idea that the enteric nervous system may be the source of the body-first subtype of PD (6). Patients with this subtype are also more likely to experience RBD during the prodromal phase. This implies that dysbiosis of the fecal microbiota could be one of the PD-RBD pathways. Furthermore, research has demonstrated that microbial alterations could hasten α-synuclein aggregation in PD patients (7). And PD patients with RBD symptoms exhibit a greater range, degree, and density of α-synuclein aggregation and abnormal deposition (8), suggesting that the microbial alterations in the PD-RBD may have started early. Currently, it is believed that fecal microbiota can also influence the differences in metabolic processes through immunological and endocrine pathways (9, 10). In particular, fecal microorganisms can affect the body’s homeostasis and normal physiological processes through the metabolites. Any disruptions in these metabolic pathways may lead to the initiation of neurological diseases. Therefore, both fecal microbiota and metabolic profiles may be involved in the development of PD-RBD. Furthermore, 82% of idiopathic RBD can develop into neurodegenerative diseases after 10.5 years (11), including PD. As the most specific prodromal marker of PD, alterations to intestinal microbiota and metabolites may also be present in iRBD. Therefore, there is a need to explore these variations to elucidate the mechanisms and thus guide the individualized treatment in PD-RBD.

In this study, we combined microbiomes and metabolomics, aiming to elucidate the characteristics and interactions of fecal microbiota and plasma metabolic characteristics of PD-RBD patients, further explore the disease pathogenesis, thus providing new insights into disease diagnosis and treatment.

## MATERIALS AND METHODS

**Fig 1:**
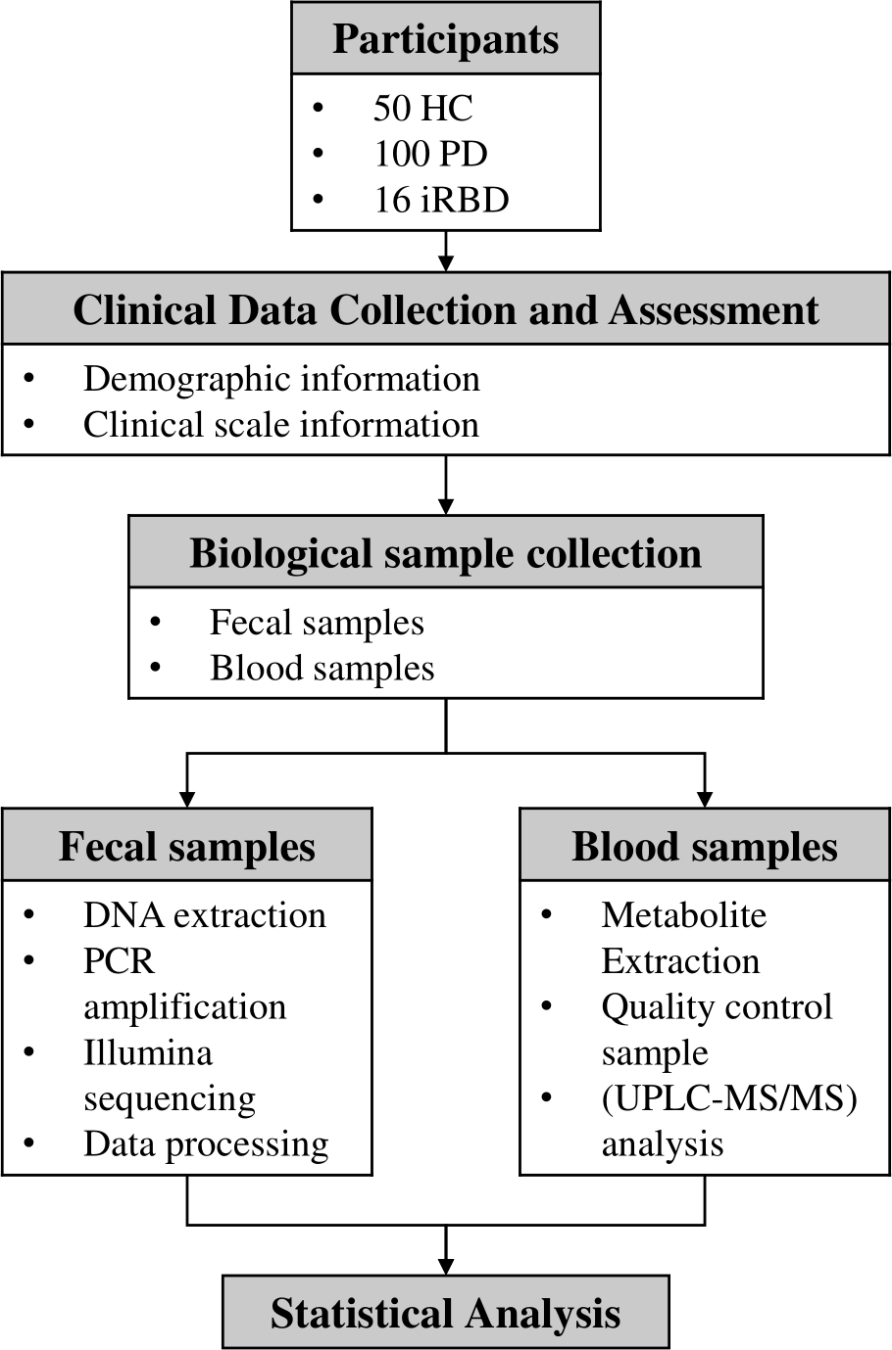
The flow chart of Materials and Methods.

The flow chart of this section is shown in Fig. 1.

### Participants

Participants were recruited at the Department of Neurology of the Affiliated Hospital of Xuzhou Medical University from May 2022 to June 2023, including 50 healthy controls (HCs) and 100 PD patients. All patients with PD satisfied the International Parkinson and Movement Disorder Society clinical diagnostic criteria in 2015 (12). We also excluded (i) patients with diagnosed psychiatric disorders; (ii) patients who are diagnosed with atypical or secondary parkinsonism; (iii) patients who take antibiotics, probiotics or prebiotics within the last 1 month; (iv) patients with co-infection; (v) patients with severe chronic acute conditions or severe cognitive impairment who could not cooperate in completing the study questionnaires; (vi) patients with an acute or chronic gastrointestinal primary disease, such as inflammatory bowel disease, irritable bowel syndrome, and so on; and (vii) patients with DBS surgery. These exclusion criteria were based on the patient’s medical history and pertinent hospitalization records. Furthermore, 16 patients with probable idiopathic RBD (iRBD) with a score of greater than or equal to 6 of the RBD Screening Questionnaire (RBD-SQ) were recruited (13). In addition, 50 HCs with no history of PD were recruited during the same period.

### Clinical data collection and assessment

Demographic information was collected from the study population including gender, age, education, height, weight, and BMI. Motor function of patients was assessed using the Unified Parkinson’s Disease Rating Scale-III (UPDRS-III) (14) and Hoehn and Yahr stage (H-Y stage). UPDRS-I was used to assess the overall nonmotor symptoms. Scale for Outcomes in PD for Autonomic Symptoms (SCOPA-AUT) was used to assess autonomic function. The Pittsburgh Sleep Quality Index (PSQI) and the Parkinson’s Disease Sleep Scale-1 (PDSS) (15) were used to assess sleep quality. REM sleep behavior disorder questionnaire-Hong Kong (RBD-HK) (16) was used to quantify the severity of RBD symptoms. More severe symptoms were indicated by a higher score on the aforementioned scales. And RBD-SQ was used to determine whether PD was accompanied by RBD, with a score of greater than or equal to 6 indicating a probable RBD in PD patients (PD-RBD) (13).

### Biological sample collection

Venous blood samples from PD patients and HCs were collected after fasting for at least 8 h, then centrifuged at 4°C to obtain plasma, dispensed, and stored at −80°C until analysis. Fecal samples were collected in sterile stool collection containers and stored at −80°C before processing.

### DNA extraction, PCR amplification, and Illumina sequencing

Total microbial genomic DNA was extracted from 150 samples using the FastPure Stool DNA Isolation Kit (Magnetic bead) (MJYH, Shanghai, China) according to the manufacturer’s instructions. The quality and concentration of DNA were determined by 1.0% agarose gel electrophoresis and a NanoDrop ND-2000 spectrophotometer (Thermo Scientific Inc., USA) and kept at −80°C prior to further use. The hypervariable region V3-V4 of the bacterial 16S rRNA gene was amplified with primer pairs 338F (5′-ACTCCTACGGGAGGCAGCAG-3′) and 806R (5′-GGACTACHVGGGTWTCTAAT-3′) (17) by an ABI GeneAmp 9700 PCR thermocycler (ABI, CA, USA). The PCR mixture including 4 µL 5× Fast Pfu buffer, 2 µL 2.5 mM dNTPs, 0.8 µL forward primer (5 µM), 0.8 µL reverse primer (5 µM), 0.4 µL Fast Pfu polymerase, 0.2 µL BSA, 10 ng of template DNA, and ddH_2_O to a final volume of 20 µL. PCR amplification cycling conditions were as follows: initial denaturation at 95°C for 3 min, followed by 27 cycles of denaturing at 95°C for 30 s, annealing at 55°C for 30 s and extension at 72°C for 45 s, and single extension at 72°C for 10 min, and end at 4°C. All samples were amplified in triplicate. The PCR product was extracted from 2% agarose gel and purified. Then quantified using Quantus Fluorometer (Promega, USA).

Purified amplicons were pooled in equimolar amounts and paired-end sequenced on an Illumina PE300 platform (Illumina, San Diego, CA, USA) according to the standard protocols by Majorbio Bio-Pharm Technology Co. Ltd. (Shanghai, China).

### Data processing

Raw FASTQ files were de-multiplexed using an in-house perl script, and then quality-filtered by fastp version 0.19.6 (18) and merged by FLASH version 1.2.11 (19). Then, the optimized sequences were clustered into operational taxonomic units (OTUs) using UPARSE 11 (20) with 97% sequence similarity level. The most abundant sequence for each OTU was selected as a representative sequence. To minimize the effects of sequencing depth on alpha and beta diversity measure, the number of 16S rRNA gene sequences from each sample was rarefied by minimum sample sequence.

The taxonomy of each OTU representative sequence was analyzed by RDP Classifier version 2.13 (21) against the 16S rRNA gene database (e.g., Silva v138) using confidence threshold of 0.7. The metagenomic function was predicted by PICRUSt2 (Phylogenetic Investigation of Communities by Reconstruction of Unobserved States) (22) based on OTU representative sequences.

### Metabolite extraction

Liquid sample (100 µL) was added to a 1.5 mL centrifuge tube with 400 µL solution (acetonitrile:methanol = 1:1 [vol:vol]) containing 0.02 mg/mL internal standard (L-2-chlorophenylalanine) to extract metabolites. The samples were mixed by vortex for 30 s and low-temperature sonicated for 30 min (5°C, 40 kHz). The samples were placed at −20°C for 30 min to precipitate the proteins. Then, the samples were centrifuged for 15 min (4°C, 13,000 × *g*). The supernatant was removed and blown dry under nitrogen. The sample was then re-solubilized with 100 µL solution (acetonitrile:water = 1:1) and extracted by low-temperature ultrasonication for 5 min (5°C, 40 KHz), followed by centrifugation at 13,000 × *g* and 4°C for 10 min. The supernatant was transferred to sample vials for LC-MS/MS analysis.

### Quality control sample

As part of the system conditioning and quality control process, a pooled quality control sample (QC) was prepared by mixing equal volumes of all samples. The QC samples were disposed and tested in the same manner as the analytic samples. This helped to represent the whole sample set, which would be injected at regular intervals (every 5–15 samples) in order to monitor the stability of the analysis.

### UPLC-MS/MS analysis

The LC-MS/MS analysis of the sample was conducted on a Thermo UHPLC-Q Exactive system equipped with an ACQUITY HSS T3 column (100 mm × 2.1 mm i.d., 1.8 µm; Waters, USA) at Majorbio Bio-Pharm Technology Co. Ltd. (Shanghai, China). The mobile phases consisted of 0.1% formic acid in water:acetonitrile (95:5, vol/vol) (solvent A) and 0.1% formic acid in acetonitrile:isopropanol:water (47.5:47.5, vol/vol) (solvent B). Positive ion mode separation gradient: 0–3 min, mobile phase B was increased from 0% to 20%; 3–4.5 min, mobile phase B was increased from 20% to 35%; 4.5–5 min, mobile phase B was increased from 35% to 100%; 5–6.3 min, mobile phase B was maintained at 100%; 6.3–6.4 min, mobile phase B was decreased from 100% to 0%; and 6.4–8 min, mobile phase B was maintained at 0%. Separation gradient in negative ion mode: 0–1.5 min, mobile phase B rises from 0% to 5%; 1.5–2 min, mobile phase B rises from 5% to 10%; 2–4.5 min, mobile phase B rises from 10% to 30%; 4.5–5 min, mobile phase B rises from 30% to 100%; 5–6.3 min, mobile phase B linearly maintains 100%; 6.3–6.4 min, the mobile phase B decreased from 100% to 0%; and 6.4–8 min, the mobile phase B was linearly maintained at 0%. The flow rate was 0.40 mL/min and the column temperature was 40°C.

### MS conditions

The UPLC system was coupled to a Thermo UHPLC-Q Exactive Mass Spectrometer equipped with an electrospray ionization (ESI) source operating in positive mode and negative mode. The optimal conditions were set as follows: source temperature at 400°C; sheath gas flow rate at 40 arb; aux gas flow rate at 10 arb; ion-spray voltage floating at −2,800 V in negative mode and 3,500 V in positive mode, respectively; normalized collision energy , 20-40-60 V rolling for MS/MS. Full MS resolution was 70,000, and MS/MS resolution was 17,500. Data acquisition was performed with the data-dependent acquisition mode. The detection was carried out over a mass range of 70–1,050 *m/z*.

### Statistical analysis

Basic demographic information analysis was performed by SPSS 23.0. The Shapiro-Wilk test was used to assess the normal distribution of the data. The continuous variables were described as mean ± standard deviation (x ± s) when normally distributed and compared between groups using the Student’s *t* test and described as median (25% quartile–75% quartile) M (P_25_–P_75_) when non-normally distributed and compared between groups using the Mann-Whitney *U* test (two groups) or Kruskal-Wallis test (>2 groups). The Kruskal-Wallis test, being a non-parametric method, is designed for comparing distributions across independent groups without assuming specific data distributions or adjusting for covariates. False discovery rate was used for post test. Categorical variables were expressed as a rate (%) and compared between groups using the chi-square test. Pearson analysis and Spearman analysis was used for correlation analysis of normal or non-normally distribution data. *P* value < 0.05 indicates that the difference is statistically significant.

Bioinformatic analysis of the fecal microbiota was carried out using the Majorbio Cloud platform (https://cloud.majorbio.com). Based on the OTUs information, rarefaction curves and alpha diversity indices, including observed OTUs, Chao1 richness, Shannon index, and Good’s coverage, were calculated with Mothur v1.30.2 (23). The similarity among the microbial communities in different samples was determined by principal coordinate analysis (PCoA) based on Bray-curtis dissimilarity using Vegan v2.4.3 package. The ANOSIM test was used to assess the percentage of variation explained by the treatment along with its statistical significance using Vegan v2.4.3 package. The linear discriminant analysis (LDA) effect size (LEfSe) (24) (http://huttenhower.sph.harvard.edu/LEfSe) was performed to identify the significantly abundant taxa (phylum to genera) of bacteria among the different groups (LDA score >3, *P* < 0.05).

The pretreatment of LC/MS raw data were performed by Progenesis QI (Waters Corporation, Milford, USA) software. At the same time, the metabolites were identified by searching database, and the main databases were the HMDB (http://www.hmdb.ca/), Metlin (https://metlin.scripps.edu/), and Majorbio. The data were analyzed through the free online platform of majorbio choud platform. Metabolic features detected at least 80% in any set of samples were retained. After filtering, minimum metabolite values were imputed for specific samples in which the metabolite levels fell below the lower limit of quantitation, and each Metabolic features were normalized by sum. To reduce the errors caused by sample preparation and instrument instability, the response intensity of the sample mass spectrum peaks was normalized by the sum normalization method, and then the normalized data matrix was obtained. Meanwhile, variables with relative standard deviation (RSD) >30% of QC samples were removed, and log_10_ processing was performed to obtain the final data matrix for subsequent analysis. Perform variance analysis on the matrix file after data preprocessing. The R package “ropls” (version 1.6.2) was used to perform principal component analysis (PCA) and orthogonal least partial squares discriminant analysis (OPLS-DA), and seven-cycle interactive validation evaluating the stability of the model. Unlike traditional partial least squares discrimination analysis (PLS-DA), OPLS-DA introduces orthogonal signal separation to enhance model interpretability and predictive performance by minimizing predictive errors rather than directly addressing covariate effects. The metabolites with VIP >2, *P* < 0.05 were determined as significantly different metabolites based on the Variable importance in the projection (VIP) obtained by the OPLS-DA model and the *P* value generated by Student’s *t* test. Differential metabolites among two groups were mapped into their biochemical pathways through metabolic enrichment and pathway analysis based on KEGG database (http://www.genome.jp/kegg/). Python package “scipy.stats” (https://docs.scipy.org/doc/scipy/) was used to perform enrichment analysis to obtain the most relevant biological pathways for experimental treatments.

## RESULTS

### Demographic and clinical characteristics of participants

The demographic and clinical characteristics of the 100 PD patients, the 50 HCs and 16 patients with iRBD are summarized in Table 1. Among the PD patients, 33 were diagnosed with PD accompanied by probable rapid eye movement sleep behavior disorder (RBD), based on RBD-Sleep Questionnaire (RBD-SQ) scores equal to or greater than 6. Regarding demographics, the differences in age (*P* = 0.001), gender (*P* = 0.013), education (*P* = 0.032), weight (*P* = 0.007), and BMI (*P* = 0.030) among these four groups were statistically significant. Concerning clinical characteristics, patients with PD-RBD had a longer duration of the disease (*P* = 0.011) and higher scores on RBD-HK (*P* < 0.001), RBD-SQ (*P* < 0.001), PDSS (*P* = 0.014), and SCOPA-AUT (*P* < 0.001) compared to those with PD without RBD (PD-nRBD). However, there were no statistically significant differences in Levodopa equivalent daily dose (LEDD) (*P* = 0.073) between PD-RBD and PD-nRBD.

**TABLE 1:** Demographic and clinical characteristics of HC, iRBD, PD-nRBD, and PD-RBD^*a*^^,^^*b*^

Characteristic	HC (*n* = 50)	iRBD (*n* = 16)	PD (*n* = 100)		Adjusted *P* value
			PD-nRBD (*n* = 67)	PD-RBD (*n* = 33)	
Age (years)	59 (55, 69)	59.81 ± 3.28	65.75 ± 1.18	69.03 ± 1.33	**0.001** ^*d*^ ^**,** *g*^
Age of onset (years)	NA	NA	60.60 ± 1.20	61.41 ± 1.55	0.692
Gender (male)	21 (42%)	13 (81.25%)	36 (53.73%)	23 (69.70%)	**0.013**
Education (years)	10 (9, 12)	9 (9,15)	9 (6,12)	7.58 ± 0.93	**0.032**
Height (cm)	164.82 ± 1.11	170.55 ± 2.13	165 (160, 170)	165 (160, 168)	0.079
Weight (kg)	66 (60, 75.5)	74 ± 3.18	63.76 ± 1.23	64.26 ± 1.82	**0.007** ^*g*^
BMI (kg/m^2^)	25.17 ± 0.45	25.36 ± 0.77	23.56 ± 0.38	23.88 ± 0.61	**0.030** ^*c*^
Disease duration (years)	NA	NA	4 (2, 8)	7 (4, 9)	**0.011** ^*e*^
LEDD (mg)	NA	NA	530.26 ± 44.50	665.86 ± 56.56	0.073
H-Y stage	NA	NA	2 (2, 3)	2.5 (2, 3)	0.607
UPDRS-I	NA	NA	12 (7, 15)	13 (10, 20)	0.063
UPRDS-II	NA	NA	13 (6, 23)	17.36 ± 1.44	0.216
UPDRS-III	NA	NA	34 (22, 57)	43.48 ± 3.02	0.286
RBD-HK	3 (0, 5)	51.43 ± 3.93	8 (0, 17)	34.48 ± 2.33	**0.000** ^*c*^ ***^,^*** ^*d* **,**^ ^*e*^ ^**,**^ ^*f*^ ^**,**^ ^*g*^ ^,^ ^*h*^
RBD-SQ	0 (0, 1)	8.36 ± 0.52	1 (0, 2)	8 (6, 9)	**0.000** ^*d*^ ^**,**^ ^*e*^ ^**,**^ ^*f*^ ^**,**^ ^*g*^
PSQI	4 (3, 6.25)	NA	10 (5, 13)	10.27 ± 0.85	**0.000** ^*c*^ ^**,** *d*^
PDSS	NA	NA	104.87 ± 3.46	89.97 ± 4.79	**0.014** ^*e*^
SCOPA-AUT	2 (1, 6)	NA	17.42 ± 1.19	25.15 ± 1.63	**0.000** ^*c*^ ^**,**^ ^*d*^ ^**,**^ ^*e*^

^*a*^ HC: healthy controls; iRBD: probable idiopathic rapid eye movement sleep behavior disorder, PD: Parkinson’s disease; PD-nRBD, Parkinson’s disease without Rapid eye movement sleep behavior disorder; PD-RBD, Parkinson’s disease with rapid eye movement sleep behavior disorder; LEDD, levodopa-equivalent daily doses; H-Y stage: Hoehn and Yahr stage; UPDRS-III, The Unified Parkinson’s Disease Rating Scale-III; RBD-HK, RBD questionnaire-Hong Kong; RBD-SQ, RBD Screening Questionnaire; PSQI, Pittsburgh Sleep Quality Index; PDSS, Parkinson’s Disease Sleep Scale-1; SCOPA-AUT, Scale for Outcomes in PD for Autonomic Symptoms.

^*b*^ Expression: normal distribution variables (mean ± SD); non-normal distribution variables (M [P_25_, P_75_]).

^*c*^ HC vs PD-nRBD.

^*d*^ HC vs PD-RBD.

^*e*^ PD-nRBD vs PD-RBD.

^*f*^ HC vs iRBD.

^*g*^ iRBD vs PD-nRBD.

^*h*^ Bold numbers indicate that the adjusted *P* value is less than 0.05.

In addition, given the small sample size of iRBD, the credibility of data results may be diminished. As a result, we focused on analyzing the PD-RBD, PD-nRBD, and HC groups in the subsequent results of fecal microbiota and metabolome, adding iRBD as an accessory element in the difference analysis.

### Fecal microbiota analysis

#### Species diversity and species composition analysis

A total of 100 PDs and 50 HCs were subjected to 16S ribosomal RNA high-throughput sequencing, and a total of 9,602,961 valid data were detected, including a total of 6,009,519 for PDs and 3,593,442 for HCs. All valid sequences were divided into OTUs, and OTUs at 97% similarity level were analyzed for bioinformatic statistics. After OTU analysis, 150 samples belonged to 17 phylums, 32 classes, 82 orders, 144 families, 382 genera, 972 species, and the total number of OUT was 5,118.

**Fig 2:**
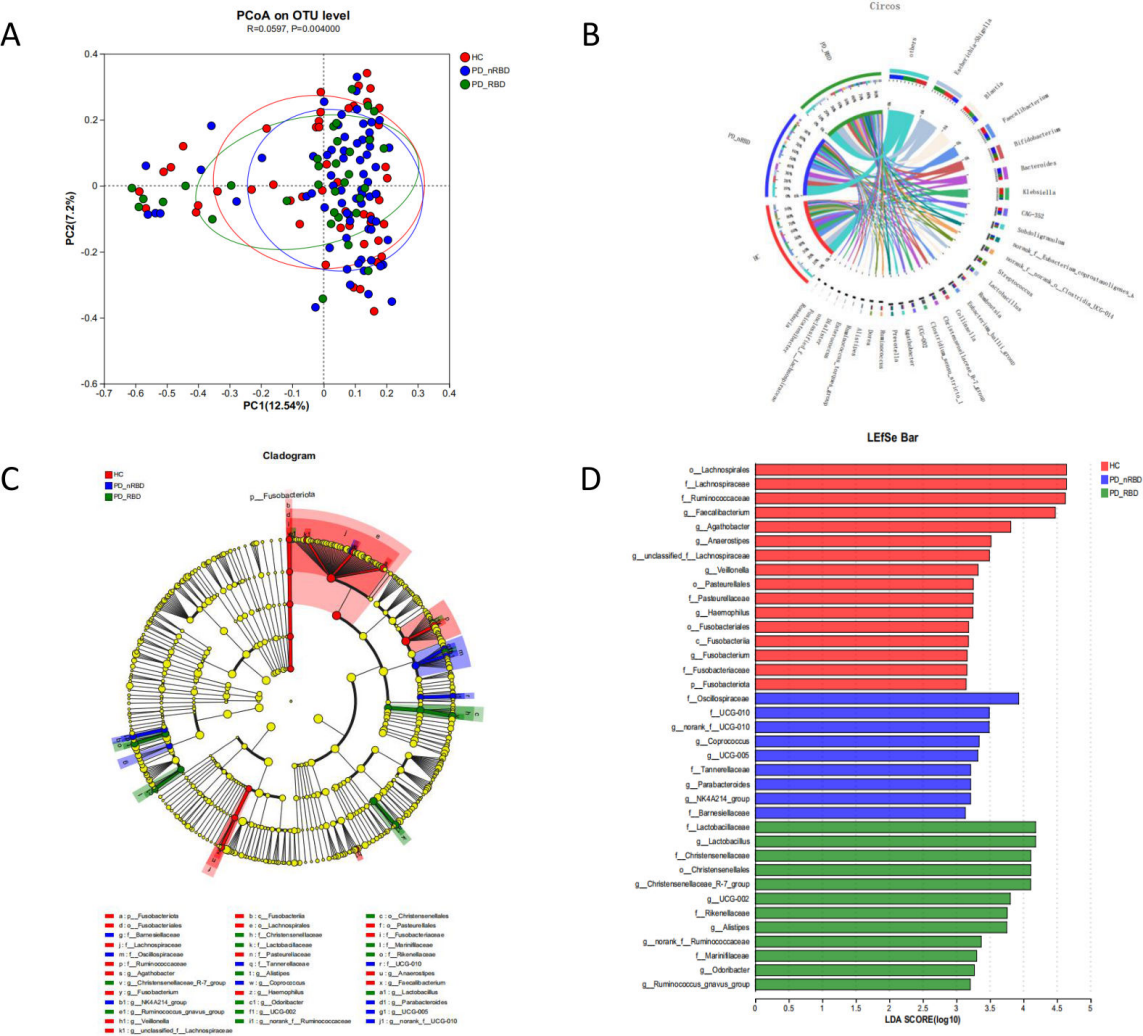
(A) Principal coordinates analysis (PCoA) diagram based on the Bray-Curtis distance. Each point on the plot represents a sample, where samples from the same group are assigned the same color. The proximity of two sample points reflects the similarity in species composition between them. (B) Visual circle diagram showing species-sample correspondence at the phylum and genus levels. (C) Lefse multilevel species hierarchical tree diagram with different colored nodes representing significantly enriched microbial taxa in their respective groups, thus contributing to intergroup differences. (D) LDA discriminant bar chart, drawn with a cutoff of 3, which indicates the relative impact of each species' abundance on the differential effect.

The differences in the following alpha diversity indices between groups were analyzed: sobs, Chao, Shannon, Simpson, and coverage. Based on the Wilcoxon rank-sum test and correction, it was found that there were no significant differences among HC, PD-nRBD, and PD-RBD groups in the above indices. Next, β-diversity was analyzed by performing PCoA based on Bray-Curtis distance. The results showed statistically significant differences among these three groups (*P* = 0.004) (Fig. 2A). We also performed a species composition analysis for these three groups. Visual circle diagrams (Fig. 2B) were used to show the species-sample correspondence at the phylum and genus levels, which helped to understand the proportion of species distribution in these three groups respectively. We can find that at the phylum level, the largest proportion of all three groups is in Firmicutes (HC: 65%, PD-nRBD: 65%, and PD-RBD: 60%). At the genus level, the largest proportion in HC was *Escherichia-Sihigella* (HC: 12%), in PD-nRBD was *Bifidobacterium* (PD-nRBD: 8.3%), and in PD-RBD was *Escherichia-Sihigella* (PD-RBD: 14%).

#### Species difference analysis and Lefse multilevel species difference discriminant analysis

To further investigate differences in microbial composition between healthy and diseased groups and identify significantly different microorganisms, we analyzed species differences at different taxonomic levels among the three groups. After correction, at the phylum level, the difference of *Fusobacteriota* among the three groups was statistically significant (*P* = 0.017). At the class level, the *Fusobacteriia* difference was statistically significant (*P* = 0.032). At the order level, a total of four species were statistically significant. Among them, the abundance of *Christensenellales* was significantly higher in PD-nRBD and PD-RBD compared to in HC group. At the family level, a total of 11 species showed statistically significant differences among the three groups. At the genus level, a total of 14 species showed statistically significant differences, including *Lactobacillus* (*P* = 0.035), *Christensenellaceae_R-7_group* (*P* = 0.035), *Fusobacterium* (*P* = 0.035), *norank_f_Christensenellaceae* (*P* = 0.037), and others. Among them, *Christensenellaceae_R-7_group* had higher levels in PD-nRBD and PD-RBD compared to the HC group. At the species level, only two differences were statistically significant after correction (Table 2).

**TABLE 2:** Abundance of differential flora between groups (%)^*a*^

Fecal microbiota at different taxonomic levels	HC (*n* = 50)	PD (*n* = 100)		Adjusted *P* value^*d*^
		PD-nRBD (*n* = 67)	PD-RBD (*n* = 33)	
Phylum				
*Fusobacteriota*	0.469 ± 3.052	0.008 ± 0.051	0.004 ± 0.014	**0.017**
Class				
*Fusobacteriia*	0.469 ± 3.052	0.008 ± 0.051	0.004 ± 0.014	**0.032**
Order				
*Christensenellales*	0.671 ± 1.392	2.388 ± 4.555	3.304 ± 6.200	**0.028** ^*b*^ *^**,**^ ^c^*
*Fusobacteriales*	0.469 ± 3.052	0.008 ± 0.051	0.004 ± 0.014	**0.028**
*Clostridia_vadinBB60_group*	0.008 ± 0.036	0.105 ± 0.607	0.025 ± 0.061	**0.011**
*Chloroplast*	0.018 ± 0.063	0.002 ± 0.004	0.002 ± 0.003	**0.047**
Family				
*Oscillospiraceae*	2.150 ± 4.580	3.758 ± 4.492	3.481 ± 4.764	**0.017**
*Lactobacillaceae*	0.917 ± 4.358	2.674 ± 7.018	3.765 ± 7.487	**0.017**
*Christensenellaceae*	0.671 ± 1.392	2.388 ± 4.555	3.304 ± 6.200	**0.017^*b,c*^**
*UCG-010*	0.083 ± 0.294	0.670 ± 1.711	0.464 ± 0.951	**0.012^*b,c*^**
*Tannerellaceae*	0.172 ± 0.579	0.498 ± 1.211	0.451 ± 1.198	**0.017**
*Fusobacteriaceae*	0.469 ± 3.052	0.008 ± 0.051	0.004 ± 0.014	**0.018**
*norank_o_Clostridia_vadinBB60_group*	0.008 ± 0.036	0.105 ± 0.607	0.025 ± 0.061	**0.012**
*unclassified_o_Enterobacterales*	0.071 ± 0.141	0.033 ± 0.085	0.015 ± 0.023	**0.033^*c*^**
*Peptococcaceae*	0.009 ± 0.032	0.041 ± 0.114	0.019 ± 0.034	**0.045** ^*b*^
*Carnobacteriaceae*	0.025 ± 0.044	0.008 ± 0.014	0.011 ± 0.034	**0.017** ^*b*^
*norank_o_Chloroplast*	0.018 ± 0.063	0.002 ± 0.004	0.002 ± 0.003	**0.033**
Genus				
*Lactobacillus*	0.916 ± 4.358	2.671 ± 7.018	3.763 ± 7.486	**0.035**
*Christensenellaceae_R-7_group*	0.665 ± 1.389	2.364 ± 4.542	3.284 ± 6.193	**0.035^*b,c*^**
*UCG-005*	0.466 ± 2.311	0.678 ± 1.418	0.495 ± 0.658	**0.033**
*norank_f_UCG-010*	0.083 ± 0.294	0.670 ± 1.711	0.464 ± 0.951	**0.033^*b,c*^**
*Parabacteroides*	0.172 ± 0.579	0.498 ± 1.211	0.451 ± 1.198	**0.033**
*norank_f_Ruminococcaceae*	0.123 ± 0.206	0.462 ± 0.696	0.518 ± 0.931	**0.033^*b,c*^**
*Fusobacterium*	0.469 ± 3.052	0.008 ± 0.051	0.004 ± 0.014	**0.035**
*UBA1819*	0.054 ± 0.207	0.150 ± 0.344	0.133 ± 0.340	**0.033**
*Butyricimonas*	0.049 ± 0.188	0.120 ± 0.287	0.075 ± 0.133	**0.037**
*norank_f_norank_o_Clostridia_vadinBB60_group*	0.008 ± 0.036	0.105 ± 0.607	0.025 ± 0.061	**0.033**
*Granulicatella*	0.025 ± 0.044	0.008 ± 0.014	0.011 ± 0.034	**0.035** ^*b*^
*norank_f_Christensenellaceae*	0.001 ± 0.005	0.011 ± 0.029	0.005 ± 0.010	**0.037** ^*b*^
*Fournierella*	0.0004 ± 0.002	0.003 ± 0.010	0.013 ± 0.039	**0.040** ^*b*^
*Paludicola*	0.001 ± 0.004	0.008 ± 0.016	0.005 ± 0.009	**0.035^*b**,^c^*^**
Species				
*uncultured_organism_g_UCG-005*	0.123 ± 0.454	0.035 ± 0.605	0.285 ± 0.442	**0.030** ^*b*^
*unclassified_g_norank_f_Ruminococcaceae*	0.021 ± 0.051	0.127 ± 0.292	0.089 ± 0.119	**0.007^*b**,^c^*^**

^*a*^ HC: healthy controls; PD: Parkinson’s disease; PD-nRBD, Parkinson’s disease without rapid eye movement sleep behavior disorder; PD-RBD, Parkinson’s disease with rapid eye movement sleep behavior disorder.

^*b*^ Expression: HC vs PD-nRBD.

^*c*^ Expression: HC vs PD-RBD.

^*d*^ Bold numbers indicate that the adjusted *P* value is less than 0.05.

Furthermore, in view of iRBD presented as a precursor to PD, stool samples from 16 iRBD patients were taken simultaneously for intestinal microbiota analysis. After correction, we discovered that the abundance of *Christensenellaceae_R-7_group* in iRBD patients was lower than in PD-nRBD patients (*P* = 0.044) and demonstrated a trend decline when compared to PD-RBD patients (*P* = 0.053), but it was moderately higher than in HC patients (*P* = 0.248). However, there were no differences in the abundance of *Lactobacillus* between iRBD and the other three groups (*P* = 0.080).

Next, Lefse’s multilevel species difference discriminant analysis was performed between these three groups and the LDA value was used to measure the magnitude of the species effect on the differential effect, suggesting that certain species may play a key role in disease. The results showed that certain significantly enriched differential species with key discriminatory effects were *Lactobacillaceae* (LDA = 4.179), *Lactobacillus* (LDA = 4.179), *Christensenellaceae* (LDA = 4.109), *Christensenellales* (LDA = 4.109), *Christensenellaceae_R-7_group* (LDA = 4.106), and others (Fig. 1C and D).

### Functional prediction

Based on 16S amplicon sequencing data, metabolic pathways showing different abundances between groups were predicted in combination with the eggNOG and KEGG databases. Combined with the eggNOG database, we found that the fecal microbiota function is mainly involved in metabolism, with amino acid transport and metabolism showing the highest abundance. In addition, translation, ribosome structure and biogenesis, carbohydrate transport and metabolism, and transcription were also identified as important components of fecal microbiota function. Similarly, 16S rRNA sequencing data combined with KEGG functional predictions suggest that fecal microbiota function is primarily related to carbohydrate metabolism, and amino acid metabolism. Additionally, deeper (tertiary) KEGG data information has allowed us to further confirm the association of bacteria with biosynthesis of secondary metabolites, microbial metabolism in different environments, and biosynthesis of amino acids.

### Metabolome analysis

In order to mitigate or minimize errors arising from the experimental and analytical processes, as well as to standardize the data structure, we appropriately processed the raw sampling data generated by instrumental analysis. This included filtering out low-quality peaks, filling in missing values, normalizing the data, evaluating the relative standard deviation (RSD) of the quality control (QC) samples, and converting the data format. Following these processing steps, a total of 2,799 metabolites were identified in both positive and negative ion modes. Specifically, 1,312 metabolites were identified in the positive ion mode, while 1,487 metabolites were identified in the negative ion mode.

### PLS-DA analysis and OPLS-DA analysis

**Fig 3:**
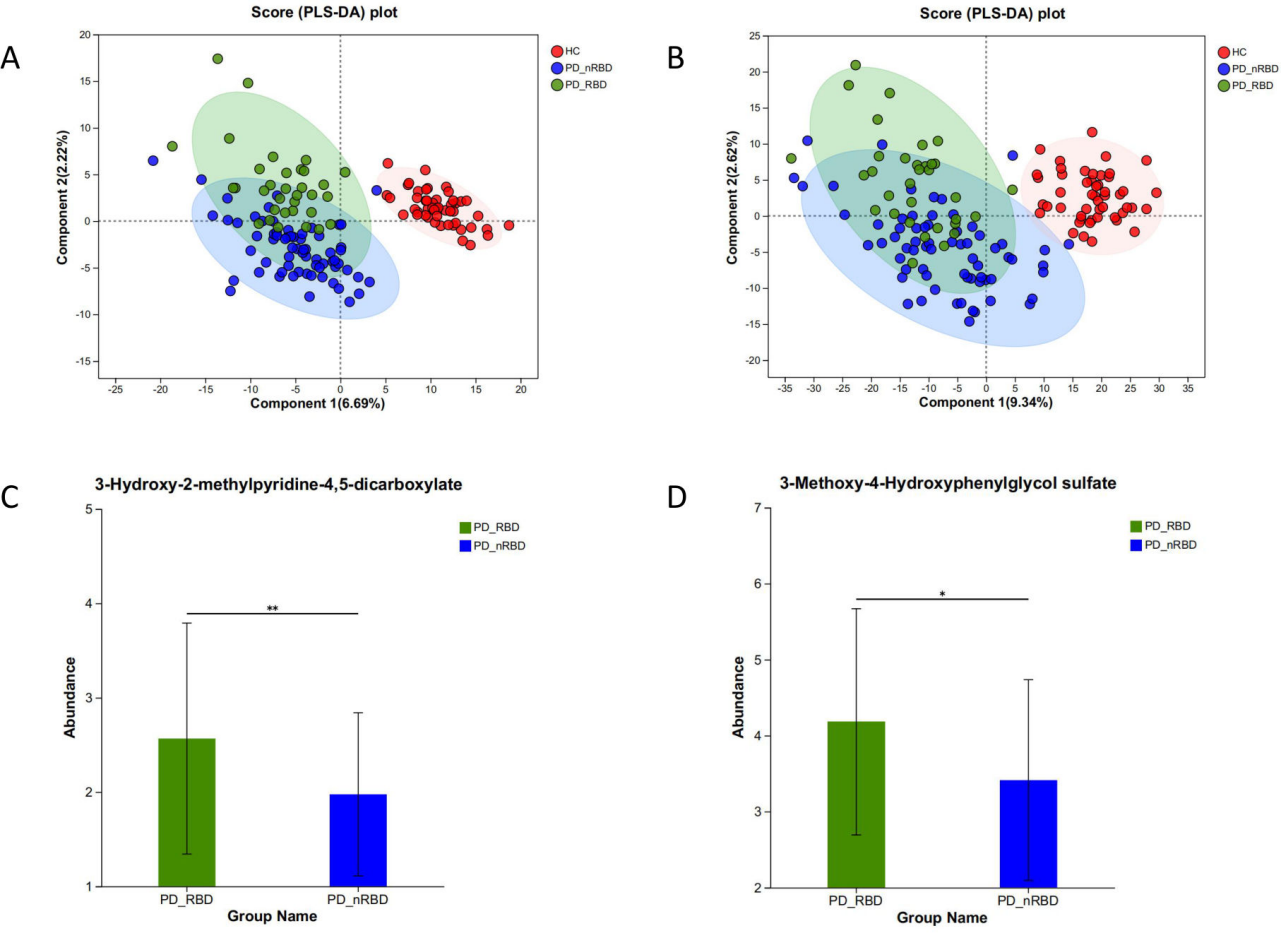
Plots of PLS-DA scores under cationic (**A**) and anionic (**B**) conditions, respectively. Greater sample separation between groups denotes a more profound classification effect. The first two principal components, Component 1 and Component 2, explain the degree of variance observed. Differential metabolite abundances in PD-nRBD and PD-RBD were shown in (C) and (D).

We employed PLS-DA to analyze the intergroup differences among the three groups of HC, PD-nRBD, and PD-RBD, which is a supervised discriminant analysis method that allows for a more precise observation of intergroup differences. Figure 3A and B presents the PLS-DA plots of the three groups in the positive and negative ion modes, which show a clear separation trend and large intergroup differences. OPLS-DA, a derivative algorithm of PLS-DA, combines orthogonal signal correction (OSC) and PLS-DA to improve the validity and parsing ability of the model. The OPLS-DA model evaluation parameters include R2X (cum) and R2Y (cum), which denote the cumulative explanatory rate of the constructed model for the X and Y matrices, respectively, and Q2, which denotes the predictive ability of the model. In the model comparing HC with PD-nRBD, R2Y = 0.979, Q2 = 0.867 in the positive mode, R2Y = 0.992, Q2 = 0.821 in the negative mode. In the model comparing HC with PD-RBD, R2Y = 0.977, Q2 = 0.897 in the positive mode and R2Y = 0.95, Q2 = 0.878 in the negative mode. This indicates that both models have a strong explanatory and predictive ability. While in the models of PD-nRBD and PD-RBD, R2Y = 0.702, Q2 = −0.040 in the positive mode, R2Y = 0.637, Q2 = −0.185 in the negative mode, indicating weak model prediction.

### Differential metabolite screening

Variable importance in projection (VIP) values based on the OPLS-DA model evaluate the importance of the role of the independent variable in explaining the dependent variable, and the larger the value, the greater the influence. In view of the weak predictive ability of the models for PD-nRBD and PD-RBD, we tightened the screening criteria to include VIP >2, *P* < 0.05, and up- and downregulated multiplicity of variance (FC) of 1.2. The results showed that a total of 78 differential metabolites were identified in the HC compared with the PD-RBD group, including 24 in the positive-ion and 54 in the negative-ion models. Furthermore, a total of nine differential metabolites were identified in the PD-nRBD compared with the PD-RBD group, including one in the positive ion mode and eight in the negative ion mode. The metabolites contributing more to the PD-nRBD versus the PD-RBD were, respectively, 3-hydroxy-2-methylpyridine-4,5-dicarboxylate (HMPD) (VIP = 5.802), lavendustin A (VIP = 5.758), 3-methoxy-4-hydroxyphenylglycol sulfate (MHPG) (VIP = 5.732), and isopropyl-beta-d-thiogalactopyranoside (VIP = 5.155) (Fig. 3C and D).

Complementary to this, we also carried out blood metabolome investigations in 16 iRBDs. We discovered that the MHPG abundances in iRBD were, respectively, higher than those in HC (VIP = 2.555) and fewer than those in PD-RBD (VIP = 3.148). Furthermore, iRBD had lower levels of HMPD (VIP = 3.314) than PD-RBD.

### Functional prediction of differential metabolites

**Fig 4:**
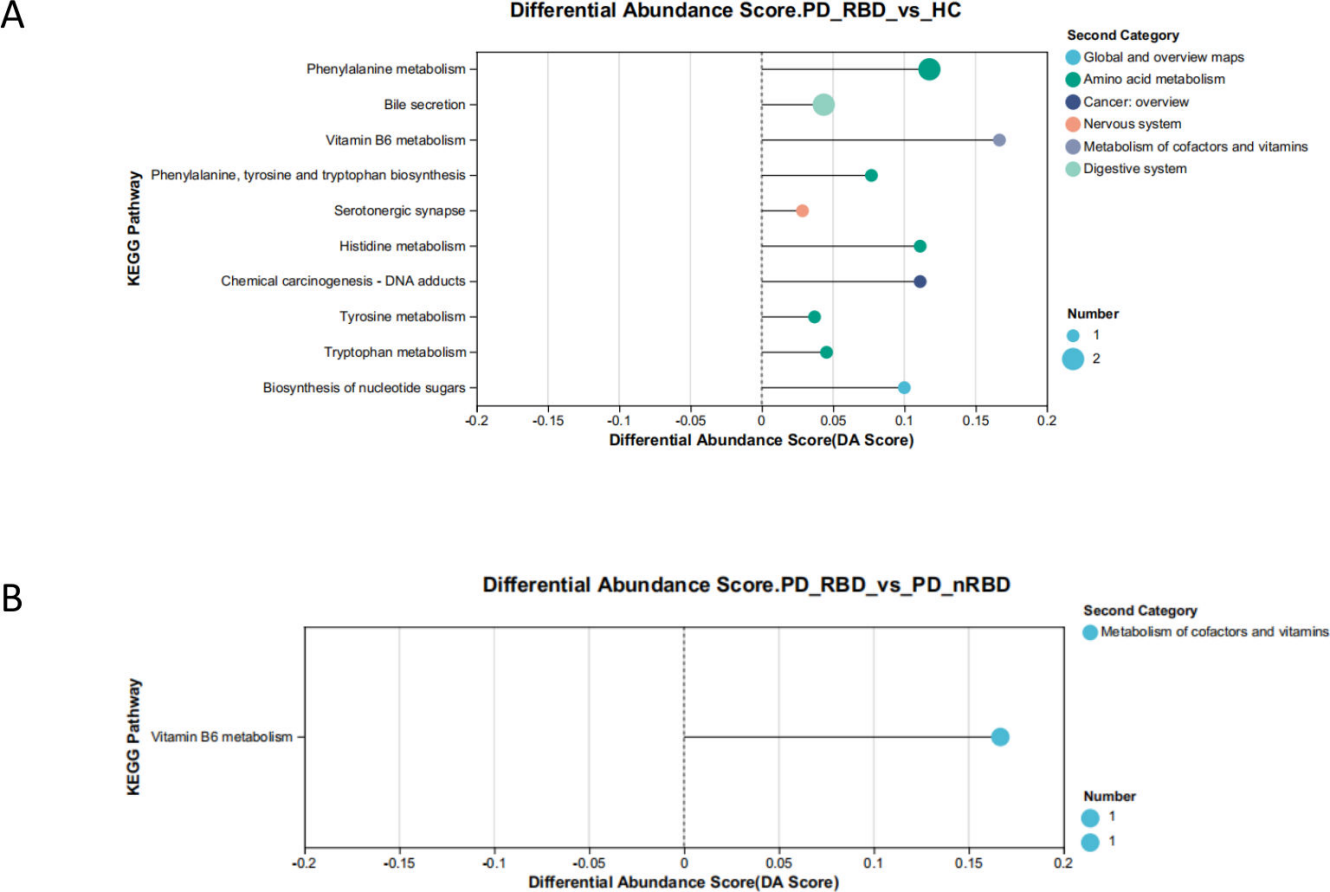
Plots of KEGG pathway differential abundance scores in HC and PD-RBD (**A**) and PD-nRBD and PD-RBD (**B**). The horizontal axis represents the differential abundance score (DA Score), while the vertical axis shows the KEGG metabolic pathway names. The length of the line segment corresponds to the absolute value of the DA score, and the dot’s size indicates the number of differential metabolites annotated in the pathway.

In this study, the types of differential metabolites detected by positive and negative modes were first combined, and then these three groups of differential metabolites were pooled together for KEGG metabolic pathway analysis. Multiple testing correction was applied to identify potential target pathways with a *P* value of less than 0.05. The KEGG pathway differential abundance score graph is shown in Fig. 4. The pathway enrichment analysis revealed that the differential metabolites of HC and PD-RBD were mostly concentrated on phenylalanine metabolism, bile secretion, and vitamin B6 metabolism. The differential metabolites of PD-nRBD and PD-RBD were mostly focused on vitamin B6 metabolism.

### Correlation analysis

#### Correlation analysis between differential species and RBD scales

**Fig 5:**
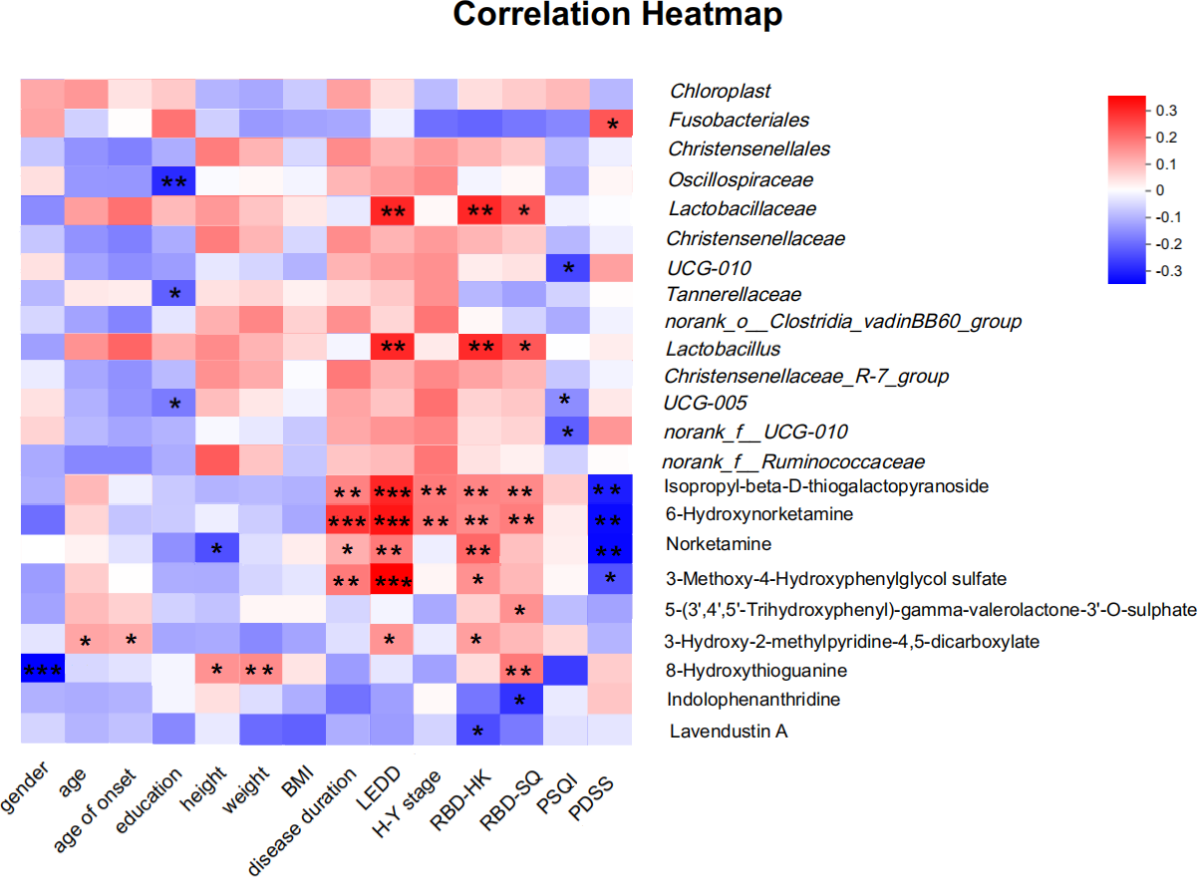
Correlation heatmap of differential species or metabolites and clinical characteristics. *P value < 0.05, **P value < 0.01, ***P value < 0.001.

Applying Spearman correlation analysis, correlation analysis was done between the differentially enriched species in PD-RBD with significant discriminatory effects and RBD-SQ as well as RBD-HK. The results were shown in Fig. 5. We found that at the order level, *Christensenellales* did not correlate statistically with both RBD-HK and RBD-SQ (*P* = 0.365, 0.516). At the family level, *Lactobacillaceae* correlated with RBD-HK and RBD-SQ (*r* = 0.270, *P* = 0.007; *r* = 0.207, *P* = 0.004). On the other hand, *Christensenellaceae* did not display a significant correlation with RBD-HK and RBD-SQ (*P* = 0.365, 0.516). At the genus level, *Lactobacillus* was correlated with RBD-HK and RBD-SQ (*r* = 0.261, *P* = 0.009; *r* = 0.200, *P* = 0.047), *Christensenellaceae_R-7_group* and *norank_f_Ruminococcaceae* were not statistically significant with RBD-HK and RBD-SQ (*P* = 0.325, 0.463; *P* = 0.874, 0.978).

#### Correlation analysis of differential metabolites with RBD scales

The correlation analysis was conducted between nine differential metabolites identified in PD-nRBD and PD-RBD and the RBD-HK and RBD-SQ scales. The findings indicated that, as Fig. 5 illustrates, several metabolites exhibited significant correlations with these scales. Specifically, 6-hydroxynorketamine (*r* = 0.258, *P* = 0.010), isopropyl-beta-d-thiogalactopyranoside (*r* = 0.2678, *P* = 0.007), norketamine (*r* = 0.317, *P* = 0.001), MHPG (*r* = 0.249, *P* = 0.013), and HMPD (*r* = 0.227, *P* = 0.023) demonstrated positive correlations with RBD-HK. On the other hand, lavendustin A (*r* = −0.211, *P* = 0.035) exhibited a negative correlation with RBD-HK. Additionally, 5-(3′,4′,5′-trihydroxyphenyl)-gamma-valerolactone-3′-O-sulfate (*r* = 0.249, *P* = 0.012), 8-hydroxythioguanine (*r* = 0.287, *P* = 0.004), 6-hydroxynorketamine (*r* = 0.281, *P* = 0.005), and isopropyl-beta-d-thiogalactopyranoside (*r* = 0.262, *P* = 0.008) showed positive correlations with RBDSQ. Conversely, indolophenanthridine (*r* = −0.252, *P* = 0.011) displayed a negative correlation with RBDSQ.

#### Correlation analysis between differential species or metabolites and other clinical characteristics

In addition to RBD scales, Fig. 5 illustrates the results of a correlation analysis carried out between fecal microbiota or metabolites and other clinical characteristics. The findings indicated a relationship between education and *Oscillospiraceae* (*r* = −0.268, *P* = 0.007), *Tannerellaceae* (*r* = −0.200, *P* = 0.046), and *UCG-005* (*r* = −0.197, *P* = 0.049). LEDD was linked with *Lactobacillus* (*r* = 0.264, *P* = 0.008) and *Lactobacillaceaehe* (*r* = 0.271, *P* = 0.006). There was no correlation between *Christensenellales*, *Chloroplast*, *Oscillospiraceae*, *Christensenellaceae*, and *norank_f_Ruminococcaceae*, with PDSS, PSQI, and demographic data (all *P* > 0.05). Furthermore, there was a correlation between MHPG and PDSS (*r* = −0.202, *P* = 0.044), LEDD (*r* = 0.481, *P* < 0.001), and disease duration (*r* = 0.286, *P* = 0.004). And HMPD was correlated with LEDD (*r* = 0.244, *P* = 0.014), age (*r* = 0.216, *P* = 0.031), and duration of disease (*r* = 0.204, *P* = 0.042). Indolophenanthridine and lavendustin A did not exhibit any correlation with PDSS, PSQI, and demographic data (all *P* > 0.05).

#### Correlation analysis of differential metabolites with intestinal microbiota

Given the potential involvement of these differential metabolites in the development of PD-RBD and their potential association with the intestinal microbiota, we selected nine differential metabolites that were screened in both PD-nRBD and PD-RBD and then combined them with the top 100 differential genera and families of bacteria that were screened for abundance in the intestinal microbiota. This allowed us to investigate the interactions between the metabolites and the intestinal microbiota. The differential metabolites and species that had a *P* value less than 0.05 are listed in Table 3. Our analysis revealed that MHPG (*r* = 0.197, *P* = 0.042) and isopropyl-beta-d-thiogalactopyranoside (*r* = 0.253, *P* = 0.011) showed a positive correlation with *Lactobacillus*. Additionally, 6-hydroxynorketamine was positively correlated with *Christensenellaceae* (*r* = 0.211, *P* = 0.035) and *Christensenellaceae_R-7_group* (*r* = 0.207, *P* = 0.038).

**TABLE 3:** Correlation of differential metabolites with differential species abundance

Differential metabolites	Differential microbiota	*r* value	*P* value^*a*^
3-Methoxy-4-hydroxyphenylglycol sulfate	*Tannerellaceae*	0.205	**0.041**
3-Methoxy-4-hydroxyphenylglycol sulfate	*Lactobacillus*	0.197	**0.049**
3-Methoxy-4-hydroxyphenylglycol sulfate	*Parabacteroides*	0.205	**0.041**
3-Methoxy-4-hydroxyphenylglycol sulfate	*norank_f_Ruminococcaceae*	0.201	**0.045**
3-Methoxy-4-hydroxyphenylglycol sulfate	*Carnobacteriaceae*	−0.232	**0.021**
3-Methoxy-4-hydroxyphenylglycol sulfate	*UBA1819*	0.259	**0.009**
5-(3′,4′,5′-Trihydroxyphenyl)-gamma-valerolactone-3′-O-sulfate	*norank_f_Ruminococcaceae*	−0.212	**0.034**
6-Hydroxynorketamine	*Christensenellaceae*	0.211	**0.035**
6-Hydroxynorketamine	*Christensenellaceae_R-7_group*	0.207	**0.038**
6-Hydroxynorketamine	*UCG-005*	0.254	**0.011**
6-Hydroxynorketamine	*Fusobacteriaceae*	−0.243	**0.015**
Isopropyl-beta-d-thiogalactopyranoside	*Lactobacillaceae*	0.258	**0.010**
Isopropyl-beta-d-thiogalactopyranoside	*Lactobacillus*	0.253	**0.011**
Norketamine	*Peptococcaceae*	0.205	**0.041**
Norketamine	*norank_o_Chloroplast*	0.208	**0.038**

^*a*^ Bold values indicate that correlation *P* value is less than 0.05.

### Receiver operating characteristic analysis

Receiver operating characteristic (ROC) analyses were carried out in HC and PD-RBD, and in PD-nRBD and PD-RBD, respectively, to ascertain the diagnostic utility of differential metabolites and differential microbiota. The differential species had a better diagnostic value for HC and PD-RBD (family level: AUC = 0.70; genus level: AUC = 0.73) than PD-nRBD and PD-RBD (family level: AUC = 0.49; genus level: AUC = 0.53). Similarly, differential metabolites had low diagnostic value in PD-nRBD and PD-RBD (AUC = 0.653) and excellent diagnostic value in HC and PD-RBD (AUC = 0.996) (Fig. S1).

## DISCUSSION

The objective of the present study was to investigate the presence of distinct microorganisms and metabolites in patients diagnosed with PD-RBD using a multi-omics approach, thus screening for the disease mechanisms. Regarding microorganisms, our study found no significant differences in the indices reflecting alpha diversity of PD-RBD, PD-nRBD, and HC, while beta diversity was statistically significant. Similar findings have been reported by previous studies (25, 26). This suggests that while the microbial community abundance and diversity of these three groups were roughly similar, the overall structural changes of the communities in the three groups varied. Furthermore, we found that differential fecal microbiota among PD-RBD, PD-nRBD, and HC encompassed *Lactobacillaceae* (*P* = 0.017), *Christensenellaceae* (*P* = 0.017), *Fusobacteriaceae* (*P* = 0.018), *Lactobacillus* (*P* = 0.035), *Christensenellaceae R-7 group* (*P* = 0.035), and *Fusobacterium* (*P* = 0.035).

The abundance of *Lactobacillus*, lactic acid-producing bacteria that are normally low in abundance within the fecal microbiota and exhibit high variability in human diseases and chronic conditions (27), was found to be elevated in patients with PD-RBD in our study, consistent with previous findings in PD and HC (28–31). Furthermore, positive correlations were observed between *Lactobacillus* and RBD-HK as well as RBD-SQ scales, indicating a potential association between *Lactobacillus* and the severity of RBD. Nevertheless, the exact role of *Lactobacillus* in the pathogenesis of PD-RBD remains uncertain. Some studies have proposed that they might exert beneficial effects by inhibiting inflammatory cytokines and exhibiting antioxidant properties, while also improving the bioavailability of levodopa (32). The increased abundance of *Lactobacillus* in PD-RBD could potentially be attributed to the enzymatic degradation of levodopa to dopamine by certain strains of *Lactobacillus*, considering that the majority of PD-RBD patients rely on levodopa-based medications (33, 34). This aligns with our finding of a favorable connection between LEDD and *Lactobacillus*. An alternative explanation could be that lactate might undergo metabolism by other bacteria, such as lactate-utilizing bacteria that do not generate butyrate, leading to the production of potentially deleterious by-products affecting intestinal health. Moreover, the increased abundance of *Lactobacillus* in PD-RBD might suggest a potential compensatory mechanism aimed at restoring intestinal homeostasis.

*Christensenellaceae* also exhibited increased abundance in PD-RBD, consistent with previous findings (28, 29, 31, 35). Previous studies have demonstrated a negative correlation between its abundance and host BMI as well as visceral fat mass (36), suggesting its potential as a marker for a lean phenotype. Therefore it could contribute to overall human health (37, 38). While our study did not reveal a significant difference in BMI between HC and PD-RBD patients, it is possible that PD-RBD patients experience greater loss of visceral and subcutaneous fat due to the higher prevalence of progressive weight loss in this population compared to HC individuals (39). According to these evidences, *Christensenellaceae* might be involved in lipid metabolism and that specific fecal microbiota increases could influence lipid absorption, which in turn could cause weight reduction. Furthermore, there was a positive correlation found between intestinal transit duration and the abundance of *Christensenellaceae* (36). This is in line with the observation that constipation is more common in PD-RBD patients than in HC individuals. Surprisingly, we discovered a similar tendency in iRBD patients. Its abundance was higher than in HC and lower than in PD-RBD, suggesting that *Christensenellaceae* alterations might have happened during the prodromal phase of PD. Furthermore, our observations indicated higher abundance of *Fusobacteriota*, and *unclassified Enterobacterales* in HC compared to PD-RBD. While the functional roles of these organisms remain unclear, our findings suggest their potential as “beneficial” microorganisms for PD-RBD.

Regarding metabolome, separate OPLS-DA models were constructed for the two groups. HMPD, MHPG, lavandarin A, and other variations are among the nine differential metabolites in PD-nRBD and PD-RBD. HMPD is a metabolite of 4-pyridoxine dehydrogenase, a vitamin B6-catalyzing enzyme (40). Its relative content was observed to be higher in PD-pRBD patients compared to PD-npRBD patients, and it showed a positive correlation with RBD-HK. Notably, functional predictions revealed that the differential metabolites in PD-RBD and PD-nRBD were associated with vitamin B6 metabolism. This implies that vitamin B6 may be connected to the occurrence of PD-RBD. Vitamin B6 has been suggested as a protective agent against Parkinson’s disease, considering that it reduces homocysteine levels (41), increases dopamine decarboxylase activity (42), and exerts anti-oxidative stress effects (43). These attributes suggest that it may hinder or retard the onset of PD. Nevertheless, a recent large study showed an association between higher vitamin B6 levels and a higher risk of PD (44). This phenomenon may result from the longer prodromal period of PD, which increases hospital visits during the initial years of diagnosis and leads to a variety of disorders being diagnosed, prompting increased self-supplementation with vitamin B6. Furthermore, low vitamin B6 levels may contribute to peripheral neuropathy (45), which is associated with autonomic dysfunction (46). PD-RBD patients typically exhibit more severe autonomic damage; which may cause a compensatory increase in vitamin B6. Therefore further relevant basic studies are necessary to confirm the mechanism of vitamin B6 in PD-RBD patients.

MHPG is a significant metabolite of central norepinephrine. The brain and spinal cord produce it and transport it to the cerebrospinal fluid and blood before excretion in urine. Studies have demonstrated that MHPG is reduced in the cerebrospinal fluid in PD (47). Damage to locus coeruleus and noradrenergic neurons may be responsible for producing the PD-RBD phenotype (48), which is similar or even greater loss than in patients with iRBD (49). Meanwhile, animal research indicating inactivation of locus coeruleus noradrenergic neurons during REM sleep (50), corroboraes this suggestion (48). Interestingly, our study found increased MHPG in PD-RBD, resembling previous AD studies (51, 52). Furthermore, we discovered that it correlated positively with disease duration and tended to rise in HC, iRBD, and PD-RBD, which may be a reflection of compensatory mechanism in surviving neurons and begin at the prodromal stage of PD. This includes over-innervation of neurons for specific brain regions, increased rates of norepinephrine synthesis and firing in damaged neurons, or reduced ability of presynaptic α2 receptors to inhibit norepinephrine activity (51, 53–55). Moreover, previous studies on MHPG may have focused on the cerebrospinal fluid in PD as the levels in the cerebrospinal fluid are more representative of the center, and few studies have explored its presence in the periphery. This suggests that central noradrenergic deficiency in PD-RBD may not be applicable in the periphery. Nevertheless, MHPG as a differential metabolite may serve as a potential biomarker for PD-RBD and pave the way for further mechanistic research.

The tyrosine kinase inhibitor lavandin A has been demonstrated to rapidly regulate dopamine transporters in rat neurons (56); however, it is yet unknown how this inhibits PD and PD-RBD. Furthermore, 6-hydroxynorketamine was found to have a favorable connection with disease duration, H-Y stage, RBD-HK, and RBD-SQ among the relationships between metabolites and clinical characteristics. 6-Hydroxynorketamine is a metabolite of ketamine that, through elevating neuronal plasticity and inducing protein synthesis, can function as an immediate and sustained antidepressant (57, 58). Additionally, by triggering the ERK/mTOR/S6 signaling pathway in the hippocampal region, Felipe C. Ribeiro has discovered that it can preserve synaptic plasticity in AD mouse models (59). We hypothesize that the compensatory elevation of 6-hydroxynorketamine in PD-RBD in our study may, in a similar way, enhance synaptic plasticity and contribute to neuroprotection in PD. However, it is unknown how other distinct metabolites work in PD-RBD, including isopropyl-beta-d-thiogalactopyranoside, which is also related to disease course, H-Y stage, RBD-HK, and RBD-SQ. To learn more about their involvement in the pathophysiology of PD-RBD, more fundamental research is required.

Since many fecal microorganisms can impact the host through metabolites, we conducted a correlation analysis. In this analysis, we observed positive correlations between MHPG and *Lactobacillus*. Liao et al. have discovered that *L. plantarum* PS128 can control neurotransmission in the dopaminergic pathway by reducing glial cell overactivation and elevating norepinephrine and neurotrophin in the PD mice model (60). This implies that the involvement of *Lactobacillus* may contribute to an increase in the relative plasma concentration of MHPG, a norepinephrine metabolite; however, more investigation is required to determine the precise production mechanism. Furthermore, we also found that 6-hydroxynorketamine was positively correlated with *Christensenellaceae* and *g_Christensenellaceae_R-7_group*. Based on our functional prediction results, this may contribute to the development of PD-RBD by affecting amino acid metabolism, especially phenylalanine metabolism, and vitamin B6 metabolism. Furthermore, an explanation could be offered by the “microbial-gut-brain axis” that has been hypothesized. Alterations in the fecal microbiota and their metabolites cause inflammation in the gut, raise intestinal permeability, make it possible for these substances to pass through the damaged intestinal barrier, and activate mucosal immune cells, which in turn causes α-synuclein to misfold and aggregate. Additionally, misfolded α-synuclein in the gut can travel from the gut to the brain through the vagus nerve. This promotes immune cell activation, causes neuroinflammation, and ultimately results in increased permeability of the blood-brain barrier, dopaminergic neuron loss, and the development of PD-RBD. However, due to the limitations of our study design, we were unable to establish a causal relationship. We attempted mediation analysis to explore this relationship, but no significant findings emerged. Thus, it remains necessary to verify whether microorganisms impact PD-RBD through metabolites in prospective studies.

The current study has several limitations. First, it is a small-scale, single-center, cross-sectional study that does not establish causal relationships despite highlighting correlations between microorganisms and metabolites, and hence there is a need for larger, longitudinal studies conducted across multiple centers. Furthermore, the determination of the presence of RBD in our study was based on the RBD-SQ scale score, which, although highly sensitive (0.842) and specific (0.962), was not confirmed by polysomnographic testing and may have been influenced by subjective factors. Finally, unmeasured factors such as diet might have affected the results, which should be considered in future studies.

### Conclusion

In summary, our study demonstrates that fecal microbiota including *Lactobacillaceae*, *Christensenellaceae*, and *Fusobacteriaceae* differ between PD-RBD, PD-nRBD, and HC. Furthermore, 3-hydroxy-2-methylpyridine-4,5-dicarboxylate and 3-methoxy-4-hydroxyphenylglycol sulfate were differential metabolites in PD-RBD and PD-nRBD. These findings may provide potential avenues for diagnosis and treatment of PD-RBD subtype, as well as mechanistic insight into its development. Further prospective studies and basic experiments should incorporate inflammatory markers and intestinal permeability to fully understand the contribution of fecal microbiota and metabolites to PD-RBD pathogenesis.

## Data Availability

The raw sequencing reads of microbiota were deposited into the NCBI Sequence Read Archive database (accession number: PRJNA1159471). The raw data of metabolome are available through the MetaboLights database (accession number: MTBLS11094).

## SUPPLEMENTAL MATERIAL

Fig. S1 msphere.00590-24-s0001.docx Receiver operating characteristic curves in different groups.

ASM does not own the copyrights to Supplemental Material that may be linked to, or accessed through, an article. The authors have granted ASM a non-exclusive, world-wide license to publish the Supplemental Material files. Please contact the corresponding author directly for reuse.
