# Supplementary material for: Alterations of fecal microbiota and plasma metabolome in patients with Parkinson’s disease with rapid eye movement sleep disorder
Source: mSphere. 2025 May 9;10(6):e00590-24. doi: 10.1128/msphere.00590-24 (PMC12188741; doi:10.1128/msphere.00590-24)
Supplement: Fig. S1 — Receiver operating characteristic curves in different groups. [file msphere.00590-24-s0001.docx]

**Supplementary material**


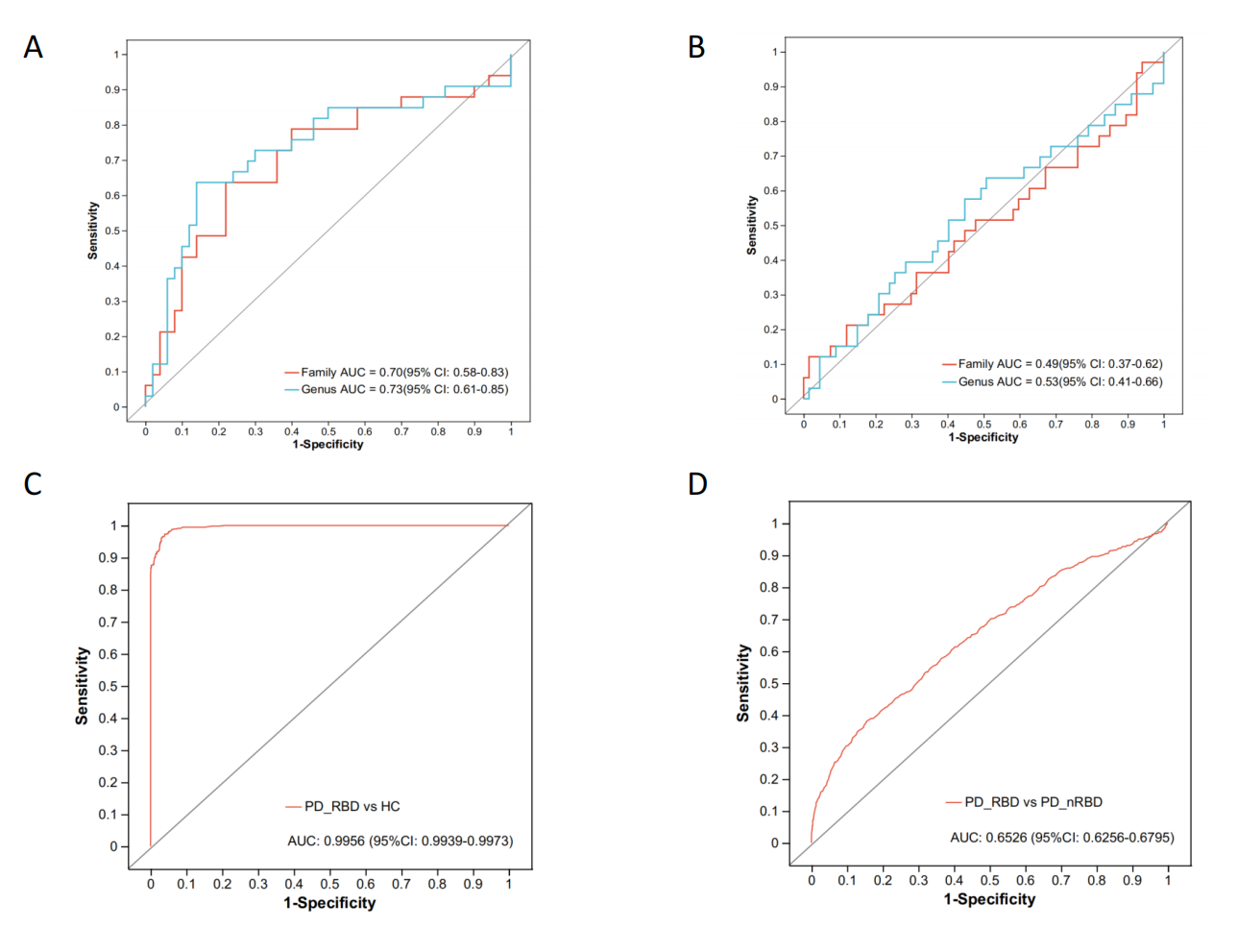


**Supplementary Fig1**

Receiver operating characteristic (ROC) curves of differential microbiota (A, B) and differential metabolites (C, D) in HC and PD-RBD, and in PD-nRBD and PD-RBD, respectively.
